# Food-borne zoonotic echinococcosis: A review with special focus on epidemiology

**DOI:** 10.3389/fvets.2022.1072730

**Published:** 2022-12-20

**Authors:** Mughees Aizaz Alvi, Abdullah F. Alsayeqh

**Affiliations:** ^1^Department of Clinical Medicine and Surgery, University of Agriculture, Faisalabad, Pakistan; ^2^Department of Veterinary Medicine, College of Agriculture and Veterinary Medicine, Qassim University, Buraidah, Saudi Arabia

**Keywords:** *Echinococcus*, prevalence, epidemiology, prevention, global scenario

## Abstract

Echinococcosis is a neglected, WHO-listed cyclozoonotic parasitic disease that is caused by a number of species belonging to the genus *Echinococcus*. This disease is widespread across the globe, resulting in heavy economic losses for farmers and cystic disease in aberrant human hosts. This review paper briefly discussed taxonomy, a brief history, the magnitude of economic losses, host spectrum and life cycle, risk factors, and clinical manifestations. Furthermore, the copro- and sero-ELISA-based prevalence of echinococcosis on different continents was summarized. Finally, the authors analyzed the frequency and use of molecular epidemiology in the taxonomy of *Echinococcus* species based on molecular markers. This review will serve as a quick reference to *Echinococcus*.

## Introduction

Infectious diseases, including parasitic infestations, are important health problems in both animals and humans (1–4), which cause economic losses and severe illness (5–10). Parasites are capable of causing acute, chronic, and debilitating types of diseases, leading to production losses in animals (11–15), and many pieces of research have shown the positive role of alternative/complementary medicine in treating parasitic diseases. Echinococcosis is a neglected silent cyclozoonotic parasitic disease caused by the metacestode stages of the genus *Echinococcus* belonging to the family Taeniidae, affecting a wide spectrum of animal species, including livestock and wildlife, and it also has zoonotic implications (16–19). The genus *Echinococcus* contains at least nine valid species with different strains and genotypes, namely *E. granulosus, E. multilocularis, E. vogeli, E. oligarthra, E. canadensis, E. equinus, E. felidis, E. shiquicus*, and *E. ortleppi*, which are important ones (20, 21).

**Figure 1:**
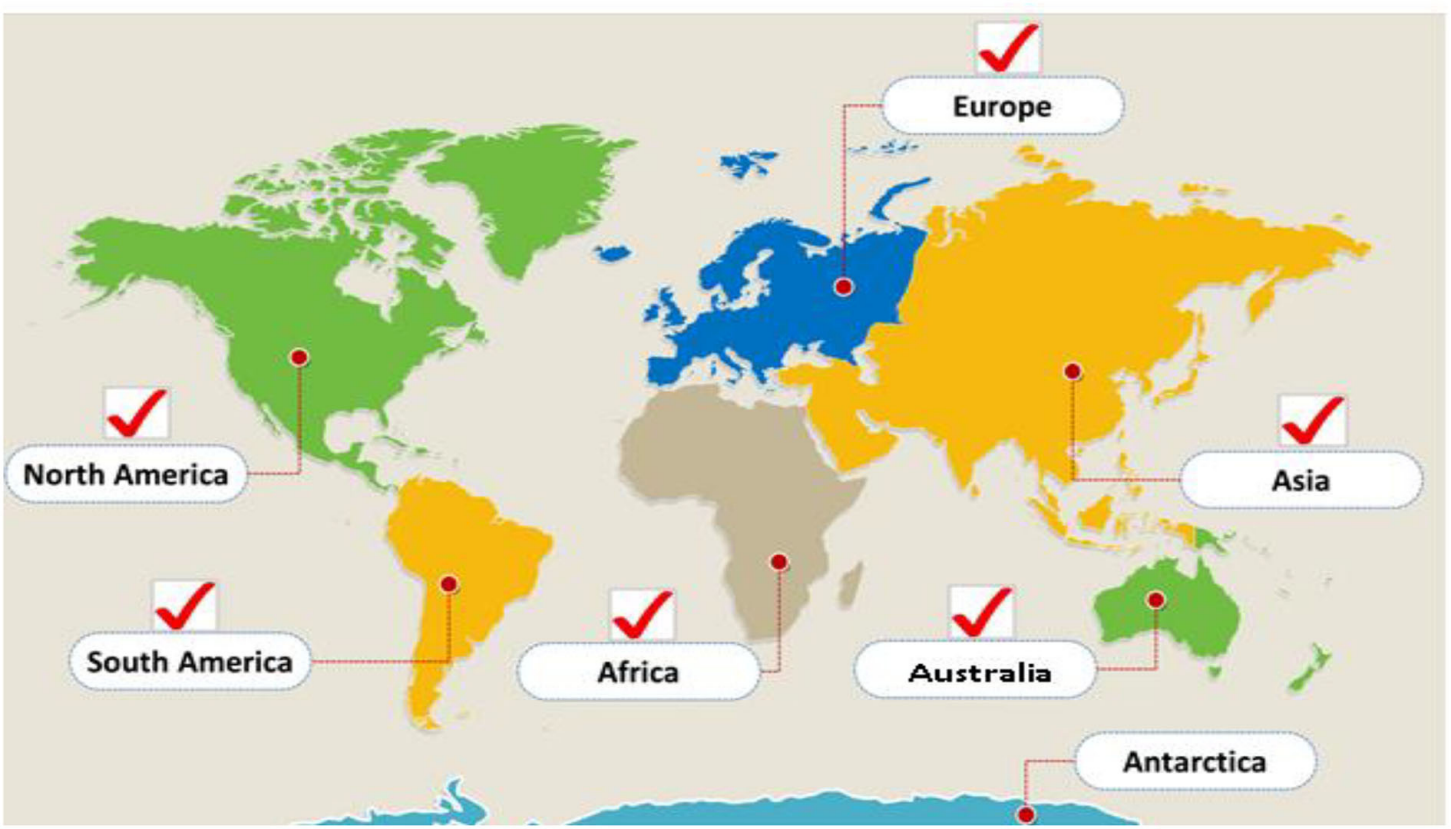
Geographical distribution of echinococcosis.

Echinococcosis is included in the World Health Organization's (WHO) list of neglected tropical diseases. This disease is widespread in its distribution and persists in a variety of environmental conditions in temperate, circumpolar, tropical, and sub-tropical regions (Figure 1). The parasite survives well in arid climatic conditions and subpolar oceanic environmental conditions. Eurasia, Australia, Africa, and South America have a very high disease prevalence, and 50 million people are infected with the disease worldwide (22, 23). Cystic echinococcosis (CE) encompasses a wide geographical area from the eastern parts of Asia to northern America and from the upper northern hemisphere to the southern countries of the African continent (24, 25).

## History

Variable-sized cysts comprise the metacestode stages of *E. granulosus*, which are filled with a transparent liquid. The presence of this clear liquid led to the coining of the term “hydatids.” A number of scientists and physicians, including Hippocrates, Galen, Aretaeus, Wolckerus, and Bonet, described the different features of hydatids in their timelines. Some researchers described them as an accumulation of serum and mucus in between the laminar cell layers. Francesco Redi first provided evidence of hydatids' metacestode nature and reported that cysticerci could move like animals (26). Jacob Hartman, a professor of medicine at the University of Königsberg/Germany, confirmed the animal-like nature of cysticerci, describing it as a small, spherical structure with a metacestode bladder. Edward Tyson, a professor at Oxford, also found the motility of the hydatids of *Cysticercus tenuicollis* to be like that of living creatures. Simon Pallas in the Netherlands described hydatids as a distinct group of bladder worms with small bodies on the inner walls of hydatids. Later, Ephraim Goeze discovered tapeworm scoleces in these small bodies called brood capsules. In 1801, Karl Rudolphi introduced the word *Echinococcus* to the world of science (27).

In the mid-nineteenth century, two different forms of echinococcosis were identified: cystic echinococcosis (CE) and alveolar echinococcosis (AE). There remained a heated debate over the aetiological agents responsible for the causation of CE and AE. Two schools of thought emerged, one with “unicasts,” believing that both forms occur due to the same species of *Echinococcus*, and the other with “dualists,” claiming that two different species are responsible for causing two different forms of the disease. However, the understanding of the dualist school of thought was supported by Professor Adolf Possely. Clear experimental proof supporting the concepts of a dualist school of thought was provided in 1928 by Posselt in his work “Der Alveolarechinokokkus und seine Chirurgie” (alveolar *echinococcus* and its surgery). He provided plausible arguments that two different species cause CE and AE, as their morphology, anatomy, and clinical signs clearly differ from each other (28).

## Economic ravages of echinococcosis

Echinococcosis is a neglected tropical disease of public health concern and has serious economic implications. Out of nine known species of *Echinococcus, E. granulosus* and *E. multilocularis* pose significant threats to human and animal health in addition to substantial economic losses (29). Treatment costs, production losses, and mortality in infected animals and aberrant human hosts are economic and social calamities caused by this infection. The cosmopolitan distribution of these ailments has led to losses of USD 3 billion annually (30). It has been reported that echinococcosis has led to annual economic losses of USD 212.35 million in India, USD 232.3 million in Iran, and USD 7.708 million in Turkey (31). Followed by *Fasciola hepatica*, CE is the most important cause of condemnation of livestock viscera. It has been estimated that in Chile, CE leads to economic losses amounting to USD 14.35 million per year (32). Echinococcosis causes substantial economic losses in Pakistan. Losses due to the disease have been estimated at USD 276.20 per 100 infected goats and sheep and USD 165.72 per 100 infected large ruminants and camels. There are losses in terms of quantity and quality of milk, wool, and meat, retarded growth, decreased fertility, and carcass condemnation (33).

## Life cycle of the *Echinococcus* species

**Figure 2:**
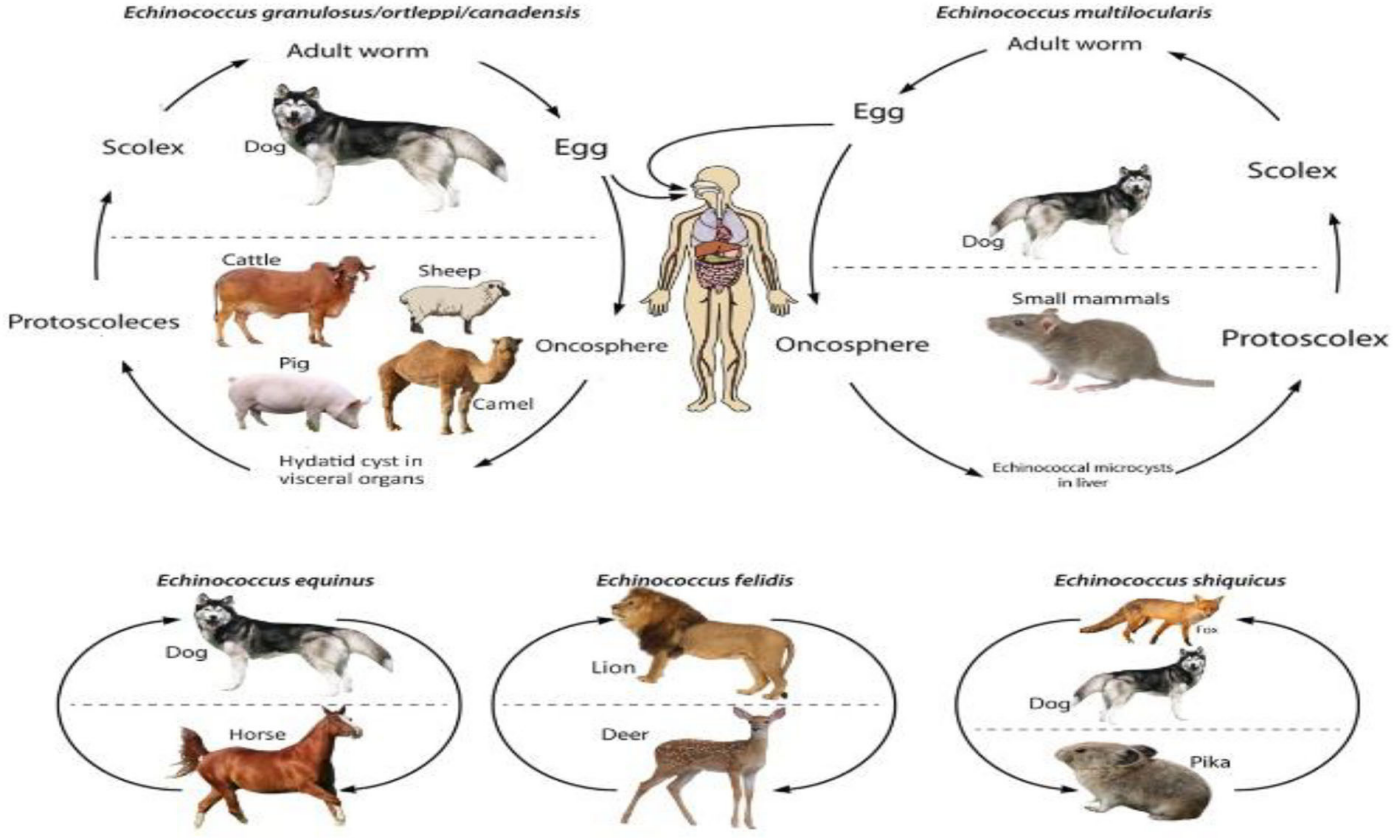
Life cycle of *Echinococcus* species.

*Echinococcus* species enjoy two different host species: the intermediate host and the definitive host. The definitive hosts of this cestode are the carnivores, especially the dogs that carry this parasite in their small intestines. Both wild and domesticated ruminants, camels, and humans serve as intermediate hosts of different *Echinococcus* species (23, 34, 35). The life cycles of different species of *Echinococcus* are depicted in Figure 2. All of the species enjoy a heteroxenic life cycle. The size of adult worms varies from 2 to 11 mm, with 2–7 proglottid segments. Scolex has two rows of rostellar hooks. Each proglottid has a single genital opening, and the mature segment is called the penultimate segment. After fertilization, eggs are fully developed in the uterus and released into the environment along with dog feces. Contaminated water or vegetation ingested by the intermediate host leads to the release of oncospheres from embryonated eggs that penetrate the intestinal wall and spread to various body tissues through circulation. Cyst formation primarily occurs in the lungs and liver, and such infected tissue, when eaten by the canids, releases protoscoleces. They develop into mature worms, completing the life cycle (36–39).

## Risk factors for cystic echinococcosis

**Figure 3:**
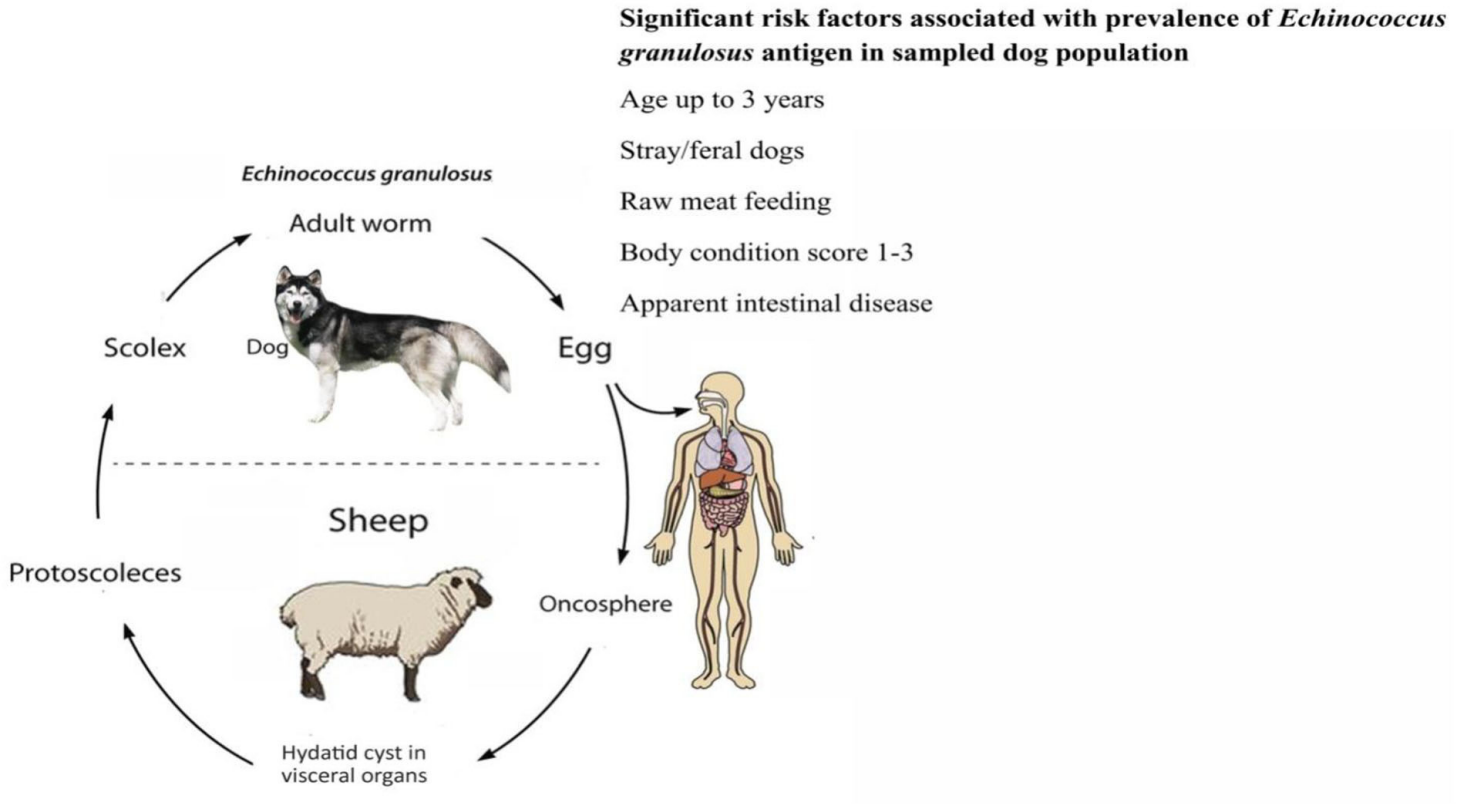
Risk factors for *Echinococcus granulosus* in dogs.

**Figure 4:**
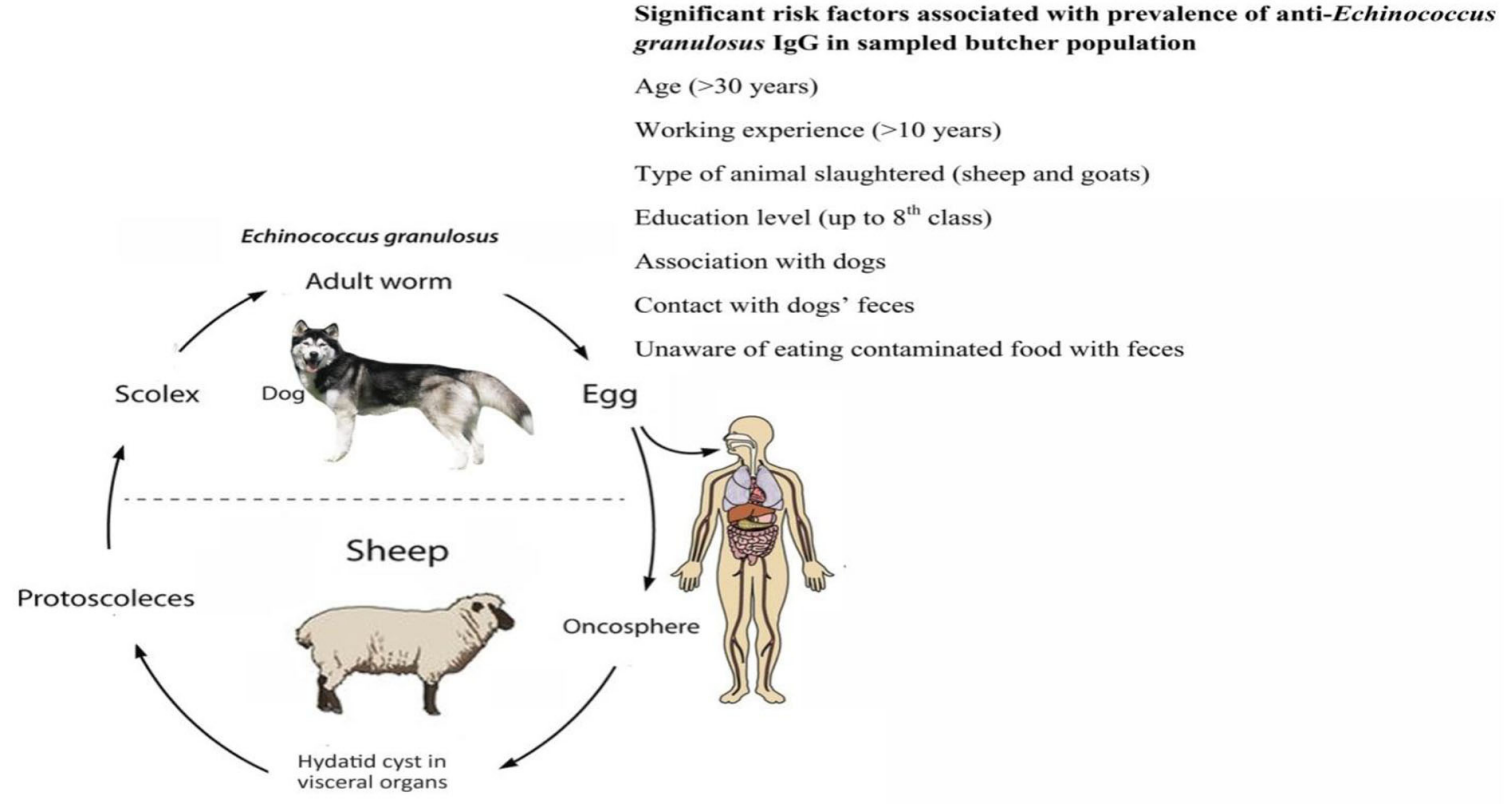
Risk factors for *Echinococcus granulosus* in butchers.

The dynamics of the completion of the life cycle of the *Echinococcus* species are well supported by the presence of dogs. Home slaughtering practices and feeding the dogs with hydatid cysts facilitate the completion of the parasite's life cycle. Younger children have a great affection for pets, including dogs. Children of such an age are likely at greater risk of suffering from echinococcosis. Regions with widespread grassland are best suited for grazing sheep, goats, and cattle. The pastoral dogs and the nomadic lifestyle play a role in the completion of the synanthropic life cycle of *E. granulosus* (40). Lack of alertness about the life cycle, poor hygiene, open slaughtering practices, close involvement of ruminants and dogs, and improper disposal of condemned carcasses/offal support the spread of echinococcosis (33). In South Asian countries like Pakistan and India, the slaughtering of food animals is carried out either in an open environment or in slaughterhouses that are easy for dogs to access. Such access by the dogs and ingestion of the offal leads to the completion of the *Echinococcus* life cycle (18). A general overview of risk factors for *E*. *granulosus* infection in dogs and humans is described in Figures 3, 4.

## Zoonotic significance in terms of DALYs

The burden of human echinococcosis can be expressed in disability-adjusted life years (DALYs), which are used to express the burden of human cases of CE and AE. The annual global burden of AE is approximately 18,200 cases, which leads to almost 666,000 DALYs (41). With regard to CE, the annual global burden amounts to 188,000 new cases every year and leads to 184,000 DALYs (42).

## Forms of echinococcosis

Echinococcosis can appear in four different forms: cystic echinococcosis (CE), alveolar echinococcosis (AE), polycystic echinococcosis (PE), and unicystic echinococcosis (UE), caused by *E. granulosus, E. multilocularis, E. vogeli, and E. oligarthra*, respectively. All of the species share the same common definitive hosts, which are the canids, except *E. oligarthra*, which has members of the family Felidae as the definitive host (43). Definitive hosts harbor adult worms in the small intestine and release the embryonated eggs of the parasite in the environment with feces. *Echinococcus granulosus* and *E. multilocularis* enjoy a wide range of intermediate hosts, including bovines, which harbor the larval stages of the parasite in their visceral organs after ingestion of the embryonated eggs (44, 45). Humans and monkeys serve as aberrant hosts for all species, and diseases may progress from asymptomatic to severe clinical diseases, which can eventually cause death (27, 46). The disease pattern of echinococcosis is usually asymptomatic in livestock, and the diagnosis is generally made on necropsy findings in the abattoir; however, it has serious implications regarding the condemnation of the carcasses. In human CE, clinical signs include asthenia, weight loss, epigastric pain, hepatomegaly, and cholestatic jaundice (47). It may become a fatal disease when the cysts rupture and their fluid contents and protoscoleces are drained into the peritoneal cavity, leading to anaphylactic shock (48).

Sequencing of mitochondrial cytochrome-c oxidase and NADH-dehydrogenase genes has revealed at least 10 genotypes (G1-G10) of CE (49). *Echinococcus granulosus sensu lato* species complex is a major veterinary and medical concern causing cystic echinococcosis (CE) in the target species across the globe. Genotyping results have indicated that *E. granulosus* sensu stricto (G1 common sheep strain, G2 Tasmania sheep strain, and G3 buffalo strain), *E. equinus* (G4 horse strain), *E. ortleppi* (G5 cattle strain), and *E. canadensis* (G6 camel strain, G7 pig strain, G8 cervid strain, G9 human strain, and G10 Fennoscandian cervid strain) are the members of this species complex (50–53). Most of the cysts are formed in the liver (70%) and the lungs (20%), while up to 10% are found in other body tissues like the brain, kidneys, marrow cavities of the bones, and ocular orbits. The chances of the formation of cysts in the central nervous system are only 1–2% of the total *E. granulosus* infections (54, 55). Apart from economic concerns, hydatidosis is a major public health issue as the hydatid cysts grow as unilocular fluid-filled bladders in the internal organs like the lungs and liver of humans (56).

## Diagnostic methods

There are a number of diagnostic techniques for echinococcosis, including ultrasonography, serological tests, e.g., enzyme-linked immunosorbent assay, immunoelectrophoresis, immunoblotting, indirect fluorescence antibody test (IFAT), and latex agglutination test (LAT), (57) and molecular methods, e.g., loop-mediated isothermal amplification (LAMP)-based techniques, and polymerase chain reaction (PCR). Each of these has its own advantages and limitations (58, 59).

For the diagnosis of *Echinococcus* infection, post-mortem analysis of the small intestine and arecoline purgation are reliable tools. Intestinal scraping, fecal sedimentation, and fecal centrifugal flotation methods have high sensitivity and are good indicators of worm burden in the host intestine. The morphology of the eggs of *E. granulosus, E. multilocularis*, and *Taenia* species resembles each other, suggesting that methods are not very sensitive and specific (60). The coproantigenic ELISA has also been another tool, as it can detect the *E. granulosus* antigen both in patent and prepatent stages. The sensitivity of this ELISA is higher for animals with a high infection burden. Another diagnostic tool to access the infection is PCR, which has high specificity and sensitivity (61). Cerebral hydatidosis is a rare form of echinococcosis and accounts for only 2% of total cases. The incidence of this form is higher in children because of frequent contact with pet dogs. However, adults can also get the infection. It is important to distinguish neurohydatidosis from other intracranial cysts when making a diagnosis. In this peculiar form, serological testing is usually not useful as far as diagnostic tools are concerned. The only reliable diagnostic technique is MRI to assess the size of the hydatid cyst and devise a plan to excise it (62) surgically.

Various techniques have been developed over the past 30 years to identify *Echinococcus* variants. However, several molecular techniques have been used in the past, such as PCR-RAPD (random amplification of polymorphic DNA), PCR-RFLP (restriction fragment length polymorphism), a Southern Blot approach, DNA fingerprinting, and dideoxy fingerprinting. PCR is the preferred method for parasite identification, molecular epidemiology research, and confirmation purposes (63).

Nested PCR and multiplex PCR are frequently used for the differential detection of different *Echinococcus* species. PCRs that are followed by sequencing help detect variations within species and genotypes (64). While sequencing is a time-consuming and skill-intensive method, it is also the most accurate method for identifying the species of *Echinococcus* and finding genetic variation.

Recently, approaches like loop-mediated isothermal amplification (LAMP) that are low-cost and simple to use have been created and tested (65). Due to the fact that DNA may be amplified using a straightforward water bath without the use of complicated instruments, LAMP is an ideal technology to utilize in low-resource settings where alveolar and cystic echinococcosis are common. However, because of its high sensitivity, this system can be tricked into giving false positives.

Contemporary methods using single-locus or multilocus microsatellite analysis have been developed to explore the genetic diversity, the population structure of parasites, and the geographic relatedness of *Echinococcus* species (66, 67).

For the identification of parasitic diseases, real-time PCR (qPCR) has a number of advantages over traditional PCR, including improved sensitivity and specificity, a shorter reaction time, and quantitative estimation of the amount of DNA in the sample (68, 69).

## Treatment and control measures

Researchers have long been interested in finding ways to break the life cycle of the Echinococcus species in order to decrease the spread of illness in both animals and people. Using anthelmintics and creating awareness among the masses about slaughter hygiene is one of the most important strategies to reduce the prevalence of echinococcosis. Limited treatment regimens are available for curing patients suffering from echinococcosis, and the focus is on giving benzimidazoles that have only parastiostatic activity. Oral administration over a long time has also led to the development of resistance to varying degrees in the metacestode stages of *Echinococcus* (47). Amphotericin-B, nitazoxanide, and isoflavones have also been reported to possess anti-*Echinococcus* activity, but the efficacy of these chemotherapeutic agents is not much higher (70). Imatinib, an anticancer agent, has been found highly effective in killing protoscoleces and metacestodes *in vitro* at a concentration of 25 μM. This drug was evaluated after keenly investigating the signaling pathways of *Echinococcus* (71). Pharmacotherapy poses different threats because of drug residues in milk and meat, which can lead to health issues for the end consumer. In addition, low dosage or frequent administration of anthelmintics leads to parasite drug resistance (36). Despite these limitations and drawbacks, vaccination remains a suitable and long-term solution against *Echinococcus* infection. Vaccine development is a crucial phase, and attention must be paid to developing multifunctional vaccines considering the circulating serotypes and genotypes of *E. granulosus* (72). Identifying the strain is key to implementing control strategies to curtail disease transmission. *Echinococcus* spp. continue to be a global problem despite our extensive knowledge of the organism, its control techniques, vaccine research, and clinical case management (73).

Eradication of this notorious infection has been a challenge for both developed and developing countries (74). However, the community's involvement in controlling the incidence of the disease is of high value. Zhang et al. proposed a number of strategies to decrease the number of future cases. Promotion and awareness seminars about the epidemiology of the disease, inspection of animal offal at the slaughterhouses, proper disposal, and condemnation of carcasses with cysts, monthly deworming of dogs with praziquantel, and ultrasonography of suspected patients and their treatment are important to highlight here (75). Killing stray dogs to prevent the shedding of embryonated eggs in the environment is another prevention strategy that was adopted in the past. A complete ban on home-slaughtering practices and rodent control programs must be institutionalized, as *E. multilocularis* targets rodents as its intermediate host (76). One alternative is to hire a Hydatid Disease Control Officer (HDCO), whose responsibilities would include registering dogs, dosing them monthly with dewormers, and educating the public about echinococcosis control programs (75).

## Prevalence of echinococcosis

### Europe

*Echinococcus granulosus* infection is endemic in Southeast European countries, e.g., Bulgaria and Romania, and *E. multilocularis* infection is hyperendemic in France, Germany, Turkey, and Switzerland (77). G1 (the sheep strain) and G4 (the horse strain) are endemic in the United Kingdom and can be found in sheep, dogs, and horse/foxhounds, respectively (78, 79). Liver lesions due to *E. multilocularis* were revealed upon necropsy of nutria (*Myocastor coypus*) in a French wildlife park. There was evidence of contamination of the interior and exterior of the park, with fox feces contacting *E*. *multilocularis*. Later, the intestines of five other foxes shot in the park were found to harbor *E. multilocularis*. A coprological analysis of other definitive hosts like wildcats, bears, and wolves showed a 5.3% prevalence (80). An epidemiological study in Turkey between 2006 and 2010 revealed that CE is endemic in Turkey.

Of the 166 buffaloes inspected after slaughter, the organs of 10.24% of the buffaloes had CE. Females and older animals had a prevalence rate of 21.66 and 37.93% compared to males and younger animals, with prevalence rates of 3.77 and 4.38%, respectively. A statistically significant difference between sex and age was observed. There were hydatid cysts in both the liver and the lungs; however, the lungs were the predominant site of encystations, with 47.06% of all cyst formation detected, compared to the liver, which had 29.41% of all cyst cases. G1, G2, and G3 genotypes were found after performing mitochondrial *cox*1 sequencing analyses. Another study was conducted in Ankara, Turkey, to compare fecal sedimentation technique and fecal centrifugal flotation to harvest taeniid eggs from the feces, identify *E. granulosus* eggs through PCR, and determine the prevalence of *E. granulosus* infection in the study area. Of 100 fecal samples, 27 had eggs. *Echinococcus granulosus*-specific PCR was carried out on the positive samples, and 14 samples were positive, resulting in a prevalence of 51.85% (50). The sedimentation technique was found to be significantly better than the flotation method, as the earlier one detected eggs in 27 samples while the latter one detected only 10 samples as positive for the eggs.

An epidemiological study was conducted in 16 counties in Romania to determine the prevalence of echinococcosis in sheep and cattle in the country. Out of 643 sheep and 1,878 cattle examined for hydatidosis, 421 sheep and 754 cattle tested positive for the cysts, giving an overall prevalence of 65.6 and 40.1%, respectively. Germinal layers were collected, and DNA was extracted to obtain an idea of the genetic diversity in the isolates. 12S ribosomal DNA and cytochrome c oxidase were used as genetic markers. The genetic diversity analysis results showed a dominance of the G1 genotype in the *E. granulosus sensu stricto* complex (81). A similar type of genetic diversity analysis was performed in Serbia to find which genotypes prevail in intermediate hosts (sheep and cattle) of the *E. granulosus sensu stricto* complex. The sequence analysis of the cytochrome c oxidase subunit 1 (*cox*1) mitochondrial gene from germinal layers of hydatid cysts showed that G2 and G3 are prevalent in the country (82).

### North and South America

In Canada, only *E. canadensis* and *E. multilocularis* have been found to be prevalent, which cause CE and AE, respectively. Both of these parasitic species were confirmed to be circulating between wild canids like wolves and foxes, which serve as definitive hosts, deer, moose, and small mammals like rodents, which serve as intermediate hosts (23, 83, 84). The USA is not endemic to echinococcosis. This infection is hard to manage and treat because of the parasite's multiorgan cyst formation potential and the high recurrence rate (85). CE has been reported in many countries in South America, including Brazil, Argentina, Chile, Peru, and Uruguay. Studies in Chile confirmed that CE is prevalent in both livestock and animals (86, 87).

### Asia

No detailed data are available with regard to the prevalence of echinococcosis in Afghanistan (88). A solitary study conducted in 1988 showed that 73% of stray dogs in Kabul harbored *E. granulosus* (89). However, CE clinical disease has been reported in Afghan immigrants and US soldiers who returned to the USA after performing duties in Afghanistan (90). Eighty percent of the Afghans work in agriculture or with animals, and many also have their own pets, like dogs. The population of stray dogs was found to be 10 times greater than that of domestic dogs. Poor law enforcement, political instability, and limited control strategies implementation are the factors for disseminated cases of CE and AE (75).

Echinococcosis is still underdiagnosed in Pakistan. Because of their limited scope, the few published reports on the disease's current state in the country cannot be considered indicative of the problem nationally. A total of 106 cases were reported to Agha Khan Hospital, Karachi, from 1995 to 2006, and 21 of them were Afghan refugees (91). The high prevalence of echinococcosis in Afghanistan and extensive free-border movement from Afghanistan into Pakistan highlight the need for conducting an extensive epidemiological study in Pakistan to assess the current status of the disease in Pakistan (92). Almost a three-decade-old study is available in the literature about the prevalence and serological investigation of echinococcosis in Islamabad, Pakistan. According to that study, hydatidosis was observed in 58.9, 38.90, and 33.06% of the slaughtered camels, cattle, and buffalo at a local slaughterhouse. Most of the cysts observed were infertile, while the specificity, sensitivity, and efficiency of indirect hemagglutination (IHA) and enzyme-linked immunosorbent assay (ELISA) were low (93).

Almost 466 million people live in Central Asian countries and are at risk of suffering from echinococcosis. Occupational exposure to farmers, herdsmen, and farmers is very high. The semi-nomadic lifestyle of the inhabitants of these areas and the raising of sheep and cattle together with dogs make the environment conducive for the *Echinococcus* species to complete their life cycle (75).

Echinococcosis is highly endemic in China, covering more than 21 provinces that comprise 87% of the total geographical area of the country. The copro-antigen ELISA, copro-DNA PCR, and necropsy of the dogs strongly suggested a high prevalence of both CE and AE in the dogs in China. As far as prevalence in cattle and sheep is concerned, CE in sheep and cattle was 50% and 44%, respectively (94).

After the fall of the Soviet Union, the number of human echinococcosis cases in Mongolia started to rise due to declining health facilities and poor dog deworming programs (95). Human CE is widely distributed in 12 provinces of Mongolia, with 10 of them adjoining Russia and China (91, 96). There is no echinococcosis control program institutionalized in Mongolia (96).

The dissolution of the Soviet Union led to the re-emergence of both human CE and AE in Kazakhstan, which are still endemic in the country due to several factors (97). Of them, nomadic lifestyles and the adoption of obsolete breeding practices shut down large farms; the establishment of small farms; the abandonment of dog deworming practices, and improper disposal of animal carcasses are important factors leading to the endemicity of echinococcosis (98).

Kyrgyzstan, Tajikistan, and Uzbekistan are endemic to human CE and AE (41, 99). The prevalence of CE in sheep and cattle in all three countries has been found to be alarming. Stray dogs and wild canines, including foxes, were found to be infected with *E. granulosus* (100). The control measures in these three countries are almost identical to those in Kazakhstan because they share similar geographical patterns, socioeconomic backgrounds, and literacy levels.

Iran is a camel-rich country with more than 1.5 million camels. CE is endemic in humans in Iran (101). Low-level endemicity was observed in northern and western Iran's sheep, goat, cattle, and camel populations (102, 103). Fecal samples from dogs and jackals were also found to be positive for containing *E. granulosus* DNA. Effective control measures were implemented in Kerman from 1991 to 1994, involving killing stray dogs and deworming pets and sheepdogs. These efforts were found to be highly effective as they decreased the incidence of the disease. An epidemiological study was conducted in five different regions of the country. Four hundred and thirty-eight dromedaries were examined, of which 135 were positive, resulting in a prevalence of up to 30.82%. The highest number of cysts were found in the lungs. The age group older than 15 years had the highest prevalence (31).

### Africa

An epidemiological study was conducted to assess the prevalence of *Echinococcus granulosus* in client-owned dogs in the Sidi Kacem province of Morocco. The reason for choosing this province was that its climatic diversity, geographic conditions, and social system make it a model representative of the country. Dogs from both rural and urban areas were included in the study. Arecoline hydrobromide was administered orally to the dogs as a purgative. Of the 273 dogs included in the study, the feces of 224 dogs had *E. granulosus* worms, resulting in an overall prevalence of 82.1%. Compared to urban-owned dogs (18.8%), dogs in rural areas had a high prevalence (38%). A comparison was made between the prevalence of echinococcosis in dogs that had access to the slaughterhouse and those that did not. Results showed that dogs with access to slaughterhouses had a higher prevalence (62.7%) as compared to dogs (29.1%) that did not have such access (104).

Apart from infecting dogs as definitive hosts and omnivores and herbivores as intermediate hosts, *Echinococcus* also infects wild animals. The first confirmed case of echinococcosis caused by *E. felidis* was reported in a lion in South Africa about 80 years ago. Molecular characterization was performed on the eggs and archived feces (105). Hyena (*Crocuta crocuta*) also serves as a definitive host for *E. felidis* (106). *Echinococcus fields* have also been found to cause echinococcosis in hippos. Six hippopotami were investigated for cysts, and three were found to have hepatic cysts. The morphology of rostellar hooks helped identify *E. felidis*, and confirmation was performed through nuclear and mitochondrial DNA sequencing (107).

*Echinococcus multilocularis* enjoys a sylvatic life cycle. The first confirmed report appeared in 2011, when a red fox was found to be the definitive host for *E. multilocularis*. This study aimed to identify rodent species that serve as intermediate hosts of *E. multilocularis*. Liver samples from 1,566 rodents were collected and examined for *E. multilocularis*-specific lesions. Samples were subjected to PCR and sequencing to identify the pathogen. Tissues were examined histologically for *E. granulosus*-specific lesions. *Microtus agrestis* (1/187), *Arvicola amphibious* (8/439), *Myodes glareolus* (0/655), and *Apodemus spp*. (0/285) had a lesion size of more than 6 mm, which is specific to *E. granulosus* (108).

The magnitude of *Echinococcus* in north African countries is widespread because of the high population of stray dogs, which consume the carcasses of infected ruminants and camels and thus take up the cysts from such condemned carcasses (109). The majority of cases of hydatidosis in Tanzania are due to the *Echinococcus granulosus* G1 genotype. This study investigated the genetic relationship between the localization of E. granulosa G1 hydatid cysts in the liver and lungs of humans, sheep, and cattle. Host species and localization differentiation were two factors responsible for genetic differentiation, as determined by single-strand conformation polymorphism and allozyme variation (110).

Eight hundred and thirty-two fecal samples from six conservation areas in Kenya were collected to determine the prevalence of *E. granulosus* in wild mammals, including jackals, hyenas, leopards, lions, and wild dogs. A total of 120 samples were positive, containing taeniid eggs, showing an overall prevalence of 14.4%. A total of 1160 eggs were collected and subjected to restriction fragment length polymorphisms—polymerase chain reaction (RFLP-PCR) of the gene *nad*1, and sequencing was performed later. Of these samples, 26 were of *E. felidis*, and 12 were of *E. granulosus sensu stricto* complex (106).

## Advancements in molecular taxonomy of *Echinococcus* species

The field of molecular epidemiology (ME) has altered our understanding of the spread of infectious diseases. This area of epidemiology provides the tools that can characterize the etiological agents of infectious illnesses. Furthermore, molecular epidemiology is useful for surveillance tasks, particularly when investigating emerging diseases. Molecular epidemiology is becoming increasingly important in the context of disease emergence and conservation for both human and domestic animal health, as well as for infectious agents in wildlife (111).

The parasite cestode *Echinococcus* is the cause of echinococcosis, which has two types—cystic and alveolar—and affects people worldwide. When investigating the epidemiology of infectious diseases, it is crucial to comprehend their etiology. It is important to understand how different host species are involved in the transmission of echinococcosis because this has been a problem in many endemic regions. This is even more important when various *Echinococcus* species, “strains,” or genotypes are present. Molecular epidemiology has been extremely helpful in understanding the wide genetic and phenotypic variability exhibited between the species present within the *Echinococcus* genus (112, 113).

Formally identifying a species requires effective communication, which is essential when the targeted species have public health significance and necessitate coordinated control measures. Regarding *Echinococcus*, there have long been taxonomic and nomenclatural ambiguities, especially at the species level. This occurred due to the lack of significant morphological features and the frequent overriding of the importance of host occurrence by taxonomic considerations (111). *Echinococcus* transmission ecology in areas with a variety of affected host species has been a topic of study for some time. Fortunately, there is widespread agreement that ten species of *Echinococcus* should be recognized based on morphological, genetic, and ecological factors. Molecular approaches usually confirm original taxonomic assumptions and, more importantly, the validity of certain morphological traits. They have also been instrumental in resolving taxonomic issues.

### Echinococcus granulosus

*Echinococcus granulosus* is the species most frequently responsible for cystic echinococcosis in humans and has the broadest spectrum of intermediate hosts (112, 113). In some regions, like Australia, it also impacts wildlife, although cycles involving cattle keep it in balance most of the time. In areas with a range of intermediate hosts that can be sensitive to other species besides *E. granulosus, E. granulosus* is also common. Because they allow for species identification from metacestode stages, molecular tools are essential in these situations. For instance, in some areas of Europe and the Middle East, livestock may contract *E. granulosus, E. ortleppi*, and *E. intermedius*, which also pose a risk to humans (112–114). It is necessary to ascertain whether distinct species cohabit in such situations to avoid impeding control strategies and focused public wellbeing measures. In many gulf countries, *E. granulosus* and *E. intermedius* are both kept sympatrically in cycles, with dogs acting as the final hosts and camelids and ovines serving as the intermediate hosts (114). Either species may occasionally infect people in mixed infestations (115). Camels are the main intermediate host for E. intermedius and, to a lesser extent, *E. granulosus* in a number of Middle Eastern regions. *E. granulosus* was the only species found in both animals and people, according to a ME investigation in Iran's Mazandaran Province. Additionally, *E. granulosus* was only found in sheep and camels, according to a ME study in Riyadh, Saudi Arabia (116). Cattle are thought to have a minor role in the spread of *E. granulosus*. However, recent investigations in Sudan and Ethiopia revealed that cattle serve as the most significant intermediate hosts for *E. granulosus, E. ortleppi*, and *E. intermedius* (117, 118).

Previous studies used host preference and phenotypic variations to discriminate between the genotypes G1, G2, and G3 of the E. granulosus specie*s* (111). Despite the need to maintain genotypic recognition in light of the phenotypic features that have epidemiological significance, current molecular characterization at a number of loci has not provided any evidence that G2 and G3 require species delimitation (119, 120).

### *Echinococcus ortleppi* and *E. equinus*

Dogs serve as the final hosts for *E. ortleppi* and *E. equinus*, whereas cattle and horses serve as the intermediate and final hosts (111). Both of these species demonstrate high host selectivity for both types of hosts. However, only *E. ortleppi* can infect humans. Dogs' high host specificity likely explains why both species are sporadic and do not have a wide geographic distribution since they rarely have access to the metacestode stages in horses and cattle, especially since public health care has progressed in the endemic regions. However, according to recent ME studies, both species are still spreading over the African and South American continents, some regions of Europe, and Turkey (121, 122). Interestingly, the first ME investigation on echinococcosis carried out in Bhutan (123) discovered that both *E. ortleppi* and *E. granulosus* were locally transmitted.

### *Echinococcus canadensis* and *E. intermedius*

It has been known for a long time that *E. granulosus* is distinct from other *Echinococcus* species and is maintained in cycles that include domestic pigs and cervids as intermediate hosts (111). According to previous ME findings (124), the four distinct *Echinococcus* genotypes (G6, 7, 8, and 10) are sustained in these life cycles. These studies added to the morphological descriptions of the adult parasites that originated in cervids and pigs. Nomenclatural issues have made it difficult to fully understand these forms' transmission cycles, despite molecular techniques' value in demonstrating the genetic distinctiveness of these forms.

There are two subspecies of the species *E. canadensis*: G8 and G10 (111). Initially, it was proposed to include the genotypes G6 and G7 in the species *E. canadensis* (125). Unfortunately, this did not reflect their ecological or geographical distribution. (124). According to molecular techniques (124, 126), the four genotypes now clearly represent two species. Sequences of G6 and G7 wildlife isolates are notably different from those of G8 and G10, and those of the latter were remarkably diverse from one another (122). Phylogenetic analysis revealed that the G6/G7 and G8/G10 groups should be considered as two discrete species, *E. intermedia* and *E. canadensis* (126). This was previously advocated (124) because the name *E. intermedius* had already been proposed for the species infecting pigs and camels.

In North America and Scandinavia, both genotypes of *E. canadensis* primarily infect wolves and other cervids, with occasional human infections (114). On the other hand, the majority of E. intermedius infections are found in the Middle East, Africa, and Europe, typically in regions where *E. granulosus* is also common. E. intermedius infections are also sustained in domestic cycles and are communicable to humans (114). The known geographic range of *E. intermedius* will likely expand as more ME surveillance is done. For instance, according to current data from various West African countries, the genotype G6 of *E. intermedius* is the most prevalent species due to the widespread use of camels (127) and is, therefore, the CE strain that poses the greatest risk to the local population's health. According to the authors of research on the illness in wild canids in Quebec and Maine (128), coyotes are more likely than wolves to contaminate urban green spaces and peri-urban habitats, according to the authors of research on the illness in wild canids in Quebec and Maine (128). This is true even though both wolves and coyotes have been shown to be hosts for *E. canadensis*. Overview about prevalent genotypes of *Echinococcus* species and molecular markers investigated in different countries and different markers used to investigate prevalence of *Echinococcus* species and corresponding number of studies covered in this review are mentioned in Tables 1, 2, respectively.

**Table 1:** Overview of prevalent genotypes of *Echinococcus* species and molecular markers that were investigated in different countries.

**Sr. No**.	**Country**	**Marker**	**Genotype**	**References**
1	Bulgaria	*nad*1	G1	(129)
2	China	*cox*1	G1 and G6	(130)
3	Italy	*rrnS*	G1 and G3	(131)
4	Chile	*cox*1	G1 and G6	(86)
5	Italy	*cox*1 *nad*1	G1, G3, G4 and G5	(132)
6	Turkey	*cox*1	G1 and G3	(133)
7	Peru	*cox*1 and *ef1a*	G1, G6 and G7	(134)
8	Iran	*cox*1	G1 and G3	(135)
9	Brazil	*cox*1 and *12S rRNA*	G1, G3 and G5	(136)
10	Iran	*cox*1 *nad*1	G1, G3 and G6	(137)
11	Australia	*cox*1, *nad*1 and *rrnS*	G1 and G3	(138)
12	Iran	*cox*1, *nad*1, *atp*6, and 12S *rRNA*	G1	(139)
13	Brazil	*cox*1	G1 and G5	(140)
14	Iran	*cox*1 and *nad*1	G1 and G3	(141)
15	Iran	*ITS*1	G1	(142)
16	Palestine	*cox*1	G1	(143)
17	China	*cox*1 and *nad*1	G1-G3 complex G6-G10 complex	(144)
19	India	*cox*1	G1, G3 and G6	(145)
20	Russia		G1, G6, G8 and G10	(146)
21	Romania	*cox*1 and 12S *rRNA*	G1 and G3	(80)
22	Chile	*cox*1 and *nad*1	G1, G3 and G4	(85)
23	Iran	*cox*1	G1, G3 and G6	(147)
24	China	*nad*2	G1	(148)
25	China	*cox*1, *cyt*b and *nad*1	G1	(149)
26	China	*cyt*b	G1	(150)
28	China	*cox*2	G1 and G6	(151)
29	Egypt	*nad*1 and *cox*1	G1, G5 and G6	(152)
30	Iran	*cox*1	G1 and G3	(153)
31	Iran	*ITS*1 and *cox*1	G1 and G6	(154)
32	Greece	*cox*1 and *nad*1	G1	(155)
33	Iran	*cox1*	G3	(156)
34	Iran	*cox*1 and *nad*1	G1, G3 and G6	(157)
35	Serbia	*cox*1	G1, G3	(81)
36	Iran	*cox*1	G1, G3 and G6	(158)
37	Bangladesh	*cox*1 and 12S *rRNA*	G1 and G3	(159)
38	Iran	*nad*1 and *cox*1	G1, G3, G5 and G6	(160)
39	Iran	*nad*1 and *cox*1	G1, G3 and G6	(161)
40	Iraq	*rrnS cox*1	G1 and G3	(22)
41	Sudan	*nad*1	G1, G5 and G6	(117)
42	Saudi Arabia	*cox*1	G1 and G3	(116)
43	Turkey		G1	(162)
44	Chile	*cox*1	G1 and G3	(163)
45	Iran	*ITS*1 and *cox*1	G1 and G3	(164)
46	China	12S *rRNA* and *cox*1	G1	(165)
47	Iran	*cox*1	G1 and G3	(166)
48	Nigeria	*nad*1 and *cox*1	G6/G7	(167)
49	Iran	*cox1*	G1 and G3	(168)
50	Iran	*nad*1 and *cox*1	G1 and G3	(169)
51	China	*cox*1	G1 and G3	(170)
52	Italy	*nad*2 and *nad*5	G7	(171)
53	Uzbekistan	*nad*1 and *cox*1	G1, G3 and G4	(172)
54	Pakistan	*nad*1 and *cox*1	G1, G3 and G5	(173)
55	Iran	*nad*1 and *cox*1	G1, and G3	(174)
56	Pakistan	*cox*1	G1, G3 and G6	(175)
57	Turkey	*cox*1	G6/G7	(176)
58	Iran	*nad*5	G1, and G3	(177)
59	Iran	*cox*1	G1	(178)
60	Mozambique	*cox*1 and *nad*1	G5	(179)
61	Iran	*cox*1 and *nad*1	G1, and G3	(180)

**Table 2:** Different markers were used to investigate the prevalence of *Echinococcus* species and a corresponding number of studies covered in this review.

**Sr. No**.	**Genetic marker**	**No. of studies**
1	*nad*1	22
2	*rrnS*	3
3	*cox*1	49
4	*ef1a*	1
5	12S *RNA*	5
6	*atp*6	1
7	*ITS*1	3
8	*nad*2	2
9	*Cytb*	2
10	16S *RNA*	1
11	*cox*2	1
12	*nad*5	2

## Conclusion

The taxonomy of *Echinococcus* has mostly been elucidated due to molecular science. As a result, a useful and educational lexicon for use in epidemiological studies has been created. We must now have the resources available to carry out epidemiological studies. These are useful in clarifying life cycles and transmission patterns in endemic areas, as previously discussed. This will be especially important in regions with several transmission cycles and the potential for mixed infections. Molecular techniques will increasingly inform and direct public health initiatives regarding clinical treatment. Those diagnosed with echinococcosis whose *Echinococcus* species of infection is unclear or who may have infections from many *Echinococcus* species are most likely to benefit. The discovery of genotypic variation within a species and its relationship with virulence foreshadows the creation of markers for clinical use.

## Author contributions

Conceptualization: MA and AA. Data curation and writing—original draft preparation: MA. Reviewing and editing: AA. Both authors contributed to the article and approved the submitted version.
